# Cut‐off values to evaluate exercise‐induced asthma in eucapnic voluntary hyperventilation test for children

**DOI:** 10.1111/cpf.12647

**Published:** 2020-06-26

**Authors:** Janne Burman, Varpu Elenius, Heikki Lukkarinen, Tom Kuusela, Mika J. Mäkelä, Olli Kesti, Katri Väätäinen, Maria Maunula, Sami Remes, Tuomas Jartti

**Affiliations:** ^1^ Skin and Allergy Hospital Helsinki University Hospital and University of Helsinki Helsinki Finland; ^2^ Department of Pediatrics and Adolescent Medicine Turku University Hospital and University of Turku Turku Finland; ^3^ Department of Physics and Astronomy University of Turku Turku Finland; ^4^ Department of Pediatrics Kuopio University Hospital Kuopio Finland

**Keywords:** asthma, bronchoconstriction, children, eucapnic voluntary hyperventilation test, exercise‐induced dyspnoea, feasibility, pulmonary function

## Abstract

**Background and Aim:**

The eucapnic voluntary hyperventilation (EVH) testing is a diagnostic tool for diagnostics of exercise‐induced bronchoconstriction; while the testing has become more common among children, data on the test's feasibility among children remain limited. Our aim was to investigate EVH testing feasibility among children, diagnostic testing cut‐off values, and which factors affect testing outcomes.

**Methods:**

We recruited 134 patients aged 10–16 years with a history of exercise‐induced dyspnoea and 100 healthy control children to undergo 6‐min EVH testing. Testing feasibility was assessed by the children's ability to achieve ≥70% of the target minute ventilation of 30 times forced expiratory volume in 1 s (FEV1). Bronchoconstriction was assessed as a minimum of 8%, 10%, 12%, 15% or 20% fall in FEV1. Patient characteristics were correlated with EVH outcomes.

**Results:**

Overall, 98% of the children reached ≥70%, 88% reached ≥80%, 79% reached ≥90% and 62% reached ≥100% of target ventilation in EVH testing; of children with a history of exercise‐induced dyspnoea, the decline percentages were as follows: 24% (≥8% fall), 17% (≥10% fall), 10% (≥12% fall), 6% (≥15% fall) and 5% (≥20% fall) in FEV1, compared to 11%, 4%, 3%, 1% and 0% among the healthy controls, respectively. Healthy controls and boys performed testing at higher ventilation rates (*p* < .05).

**Conclusion:**

Eucapnic voluntary hyperventilation testing is feasible among children aged 10–16 years and has diagnostic value in evaluating exercise‐induced dyspnoea among children. A minimum 10% fall in FEV1 is a good diagnostic cut‐off value. Disease status appears to be important covariates.

## 1 INTRODUCTION

Exercise‐induced dyspnoea is a subjective experience of breathing discomfort during exercise (Weatherald, Lougheed, Taille, & Garcia, 2017) and affects around 14% of school‐age children (Johansson et al., 2018). The two primary reasons for exercise‐induced dyspnoea include exercise‐induced bronchoconstriction (EIB) and dysfunctional breathing (Johansson et al., 2015; Cichalewski et al., 2015; Depiazzi & Everald, 2016). Dysfunctional breathing can be defined as alteration in the normal patterns of breathing (Depiazzi & Everard, 2016), and the typical manifestations of DFB are vocal cord dysfunction and hyperventilation. The prevalence of the former is 5%–20% (Cichalewski et al., 2015; Johansson et al., 2015; Tilles, 2015), and the latter is 6%–8% among school‐age children (de Groot, 2011; Johansson et al., 2015). The proper diagnosis is important because the treatments of these conditions are quite different.

The American Thoracic Society (ATS) has recommended that EIB should be diagnosed by establishing changes in lung function provoked by exercise (Parsons et al., 2013). The eucapnic voluntary hyperventilation (EVH) test is an alternative method to other indirect or direct bronchial challenge tests such as exercise challenge or methacholine challenge test that has been described as a sensitive technique for diagnosing EIB (Anderson, Argyros, Magnussen, & Holzer, 2001; Dickinson, McConell, & Whyte, 2011). The EVH test has traditionally been used for elite athletes (Anderson et al., 2001; Dickinson et al., 2011) and is widely regarded as the gold standard tool for assessing EIB among athletes (Hull, Ansley, Price, Dickinson, & Bonini, 2016). A minimum 10% fall in forced expiratory volume in 1 s (FEV1) is generally considered significant (Hallstrand et al., 2018; Parsons et al., 2013). There is only one large scale study in ordinary adults (Brummel, Mastronarde, Rittinger, Philips, & Parsons, 2009), where 71% of adults reached minimum 70% of target minute ventilation, meaning 60% of maximal minute ventilation. In total, 28% of the study patients with asthma‐like symptoms had 10% fall of FEV1%. On the other hand, 44 of 224 (20%) non‐symptomatic adult elite athletes had minimum 10% fall of FEV1 after EVH (Price et al., 2016). According ERS specificity is higher with criterion of minimum fall of 15% FEV1 compared cut‐off 10% (Hallstrand et al., 2018). EVH can also provoke vocal cord dysfunction (Christensen & Rasmussen, 2013; Turmel, Gagnon, Bernier, & Boulet, 2015).

Although the EVH test is standardized, very few studies to date have examined such testing among children. Previous studies have shown that subjects generally tolerate EVH testing (Chateaubriand do Nascimento Silva Filho MJ, 2015; Kirkby et al., 2015), but only a minority of children can reach the target minute ventilation during testing (Chateaubriand do Nascimento Silva Filho et al., 2015; Van der Eycken et al., 2016).

EVH testing is becoming more common among children for the diagnostics of exercise‐induced dyspnoea, but the data remains scarce. In this study, we thus aimed to explore the feasibility of EVH testing among children with exercise‐induced dyspnoea. We hypothesized that EVH testing would be feasible among children aged 10–16 years and that the test could provoke bronchoconstriction among children who experience exercise‐induced dyspnoea. We also wanted to determine whether a cut‐off value of a 10% fall in FEV1 could be used among children, much as such a cut‐off value is recommended for adults. Finally, we have investigated whether patient characteristics might influence testing outcomes.

## 2 MATERIAL AND METHODS

### 2.1 Recruitment

The study was conducted at the paediatrics departments of the university hospitals of Turku and Kuopio, Finland. The inclusion criteria included a suspicion of pathological reasons for exercise‐induced dyspnoea, exercise‐induced bronchoconstriction or dysfunctional breathing in patients between 10 and 16 years. The exclusion criteria were physical inactivity, severe comorbidity or chronic autoimmune disease. The 100 healthy controls, from the same age range, were recruited through local sports clubs. Their inclusion criteria included engaging in sporting activity without symptoms of exercise‐induced dyspnoea, active asthma or severe comorbidity. The Ethics Committee of the Hospital District of Southwest Finland approved the study, and written informed consent was provided by all the participants and their guardians.

### 2.2 Background data

The Childhood Asthma Control Test was completed by the participants and their guardians (Liu et al., 2007). In addition, a written questionnaire, completed by the guardian, was used to collect information about the subjects' previous medical history including doctor diagnosed asthma ever, current sporting activity, allergies, and acute and chronic respiratory symptoms, including cough, exercise‐induced dyspnoea, running nose, fever and throat symptoms.

### 2.3 Testing prerequisites

Beta_2_‐agonists were not administered for 12 hr before the tests. The baseline FEV1 had to be at least 70% of the age‐ and height‐related reference values (Koillinen, Wanne, Niemi, & Laakkonen, 1998). If a patient had an acute respiratory infection, then the test was postponed for 2 weeks.

### 2.4 Flow‐volume spirometry

The test began with baseline spirometry in which FEV1 was the main outcome (Moore, 2012). The subjects then underwent EVH testing, as described below, and spirometry was repeated 1, 5 and 10 min after the test. Finally, patients were given 0.4 mg of salbutamol in the form of a dry powder at the Turku Centre (Buventol Easyhaler: Orion Pharma) and as a spray at the Kuopio Centre (Ventoline Evohaler: Glaxo Wellcome Production) with a Babyhaler spacer device (Glaxo Wellcome Production), based on each centre's routine clinical practice. The spirometry test was repeated 15 min after the administration of salbutamol. During bronchodilatation testing, an improvement of 12% or more in FEV1 compared to baseline was interpreted as significant (Pellegrino et al., 2005).

### 2.5 Eucapnic voluntary hyperventilation test

**FIGURE 1:**
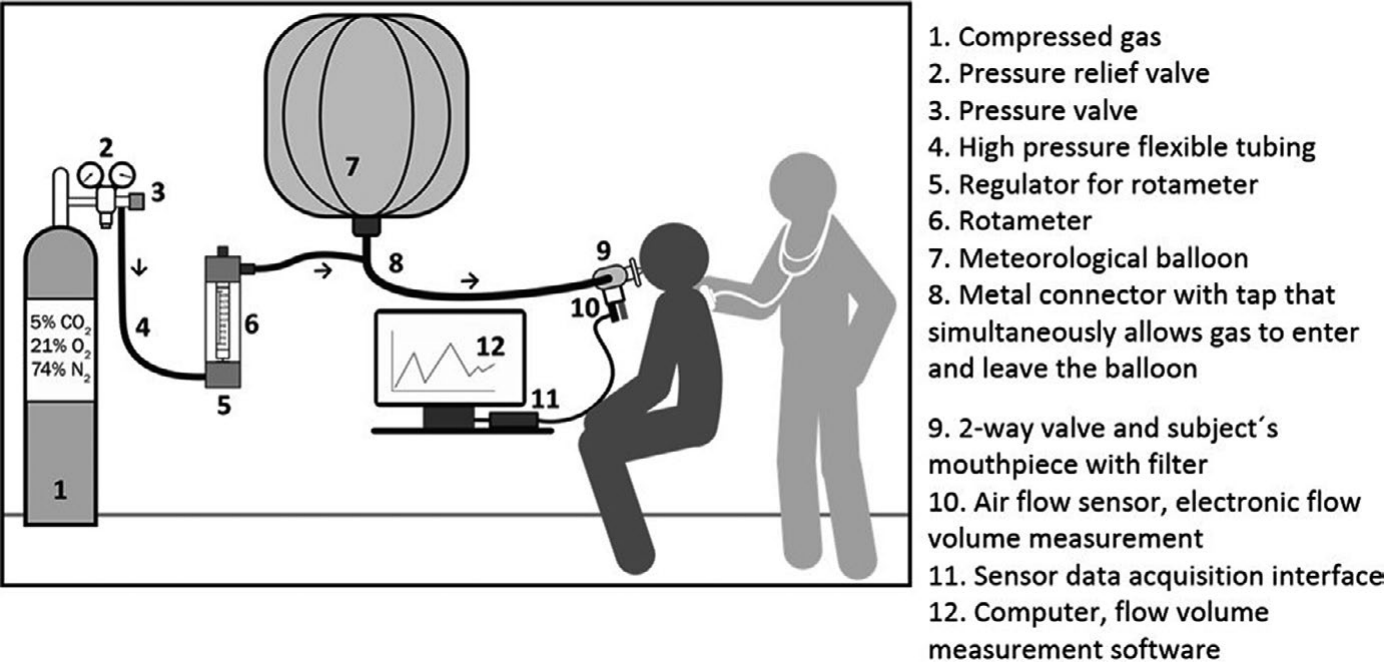
EVH test equipment

The duration of the EVH test was 6 min (Anderson et al., 2001; Burman et al., 2018). The test equipment is shown in Figure 1. The test involves the patient inhaling gas that contains oxygen plus 74% nitrogen and 5.1% carbon dioxide. The target minute ventilation in the EVH test was defined as 30 times each patient's baseline FEV1, which was equivalent to 85% of minute ventilation volume (Hallstrand et al., 2018; Parsons et al., 2013). The mouthpiece used was MicroGard II Bacterial/Viral Filter kit (ref V‐892380), Vyaire Medical Inc. The feasibility of the EVH test was assessed by the ability of participating children to achieve the minimum 70% target level of the minute ventilation volume (Hallstrand et al., 2018; Parsons et al., 2013). Minute ventilation was measured using the mouthpiece airflow sensor in real time (Burman et al., 2018). The software (WinCPRS, Absolute Aliens Oy) used in the EVH test equipment allowed us to show in real time the continuous 10‐s sliding average of the minute ventilation on the monitor graphically with the specific target level‐line that allowed the study subject to maintain the targeted minute ventilation. At the end of the test, all ventilation data were saved, and the average minute ventilation was calculated for the 6‐min examination time.

### 2.6 Definitions of exercise‐induced asthma and dysfunctional breathing

Exercise‐induced asthma was defined if study physician suspected asthma and if objective proof of bronchial hyperresponsiveness (either 10% fall of FEV1 in EVH or free running test if was made, or minimum 12% improvement of FEV1 in bronchodilatation test compared baseline value or exhaled nitric oxide (FeNO) minimum 35 ppb if was made).

Dysfunctional breathing was defined if objective symptoms such as inspiratory stridor, hyperventilation or other abnormalities of breathing occurred without bronchoconstriction during the EVH test (Depiazzi & Everard, 2016).

### 2.7 Outcomes

The primary aim of this study was to investigate the feasibility of EVH testing by assessing whether participants could achieve ≥70% of the target minute ventilation; additional target levels of ≥80%, ≥90% and ≥100% were also analysed. The second aim was to evaluate whether a guideline‐based cut‐off value of a 10% fall in FEV1 (Hallstrand et al., 2018; Parsons et al., 2013) provoked by hyperventilation could differentiate cases from controls; additional target levels of 8%, 12%, 15% and 20% fall in FEV1 were also analysed. The third aim was to determine whether common patient characteristics, age, sex, current physician‐diagnosed asthma, Childhood Asthma Test score (Liu et al., 2007), current atopic eczema, baseline FEV1, achieved minute ventilation level (70%–99% vs. ≥100% level) or response to bronchodilator correlated with the EVH outcomes.

### 2.8 Statistical analysis

SPSS version 22 (IBM Corp.) was used for the statistical analysis. For continuous parametric and non‐parametric data, Student's *t* test and the Mann–Whitney *U* test or Kruskal–Wallis tests were used, respectively. For categorical data, the chi‐square test, Fisher's exact test (when counts were <5) and McNemar's test (related samples) were used. Logistic regression analysis was used to adjust for age and sex during analyses of target minute ventilation between groups. The statistical significance was established at *p* < .05.

## 3 RESULTS

### 3.1 Study population

We enrolled 234 children; of these, 134 were cases with a history of exercise‐induced dyspnoea, and 100 were controls without any exercise‐induced symptoms.

### 3.2 Subject characteristics

The mean age of the 234 children was 13.7 years (standard deviation [*SD*] 1.9 years), and the mean baseline FEV1 was 96% of the predicted normal level (Table 1). Girls achieved a greater per cent predicted FEV1 than boys: (98.7% [*SD* 11.0] vs. 94.0% [*SD* 11.6]; *p* = .002). Cases with a history of exercise‐induced dyspnoea were more often girls than among the controls (57% vs. 29%; *p* < .001), had more atopic eczema (33% vs. 19%; *p* = .018), more often had physician‐diagnosed asthma (33% vs. 5%; *p* < .001) and had lower Childhood Asthma Test scores than the healthy controls (mean 21.1 vs. 26.2 points; *p* < .001) (Table 1). The controls participated in competitive sporting activities more than the patients (*p* < .001), possibly due to fact that they were recruited from sports groups. No other differences in characteristics were found between the subjects (Table 1).

**TABLE 1:** Baseline characteristics of the children

	Cases *n* = 134	Controls *n* = 100	*p*‐value
Age (years)	13.8 (1.8)	13.7 (2.9)	.68
Gender (boys)	58 (43%)	71 (71%)	**<.001**
Previous asthma: doctor diagnosed	42 (33%)	5 (5.0%)	**<.001**
Atopic eczema: doctor diagnosed	42 (31%)	19 (19%)	**.018**
Current parental smoking	21 (16%)	14 (14%)	.71
Regular current sports club activity	113 (84%)	100 (100%)	**<.001**
FEV1, per cent predicted	95.4 (11.8)	97.0 (11.3)	.27
Childhood asthma test score (points)	21.1 (3.4)	26.2 (1.6)	**<.001**

Note

Bold means *p* < .05. Data represent the means and standard deviations or the number of children and the percentage. FEV1: forced expiratory volume in 1 s. Independent samples *t* test, chi‐square test and Fisher's exact test were used for statistics.

### 3.3 Ability to maintain target minute ventilation during eucapnic voluntary hyperventilation testing

Of all 234 children, the minimum 70% of target minute ventilation was achieved by 229 (98%), ≥80% by 207 (88%), ≥90% by 185 (79%) and ≥100% by 144 (62%) of subjects. The boys achieved a minimum 80% of the target minute ventilation (120 [93%] vs. 87 [83%]; *p* = .015) and the minimum 90% of target minute ventilation (112 [87%] vs. 73 [70%]; *p* = .001) better than girls. None of the other patient characteristics were associated with the target minute ventilation level the subjects achieved (data not shown).

**FIGURE 2:**
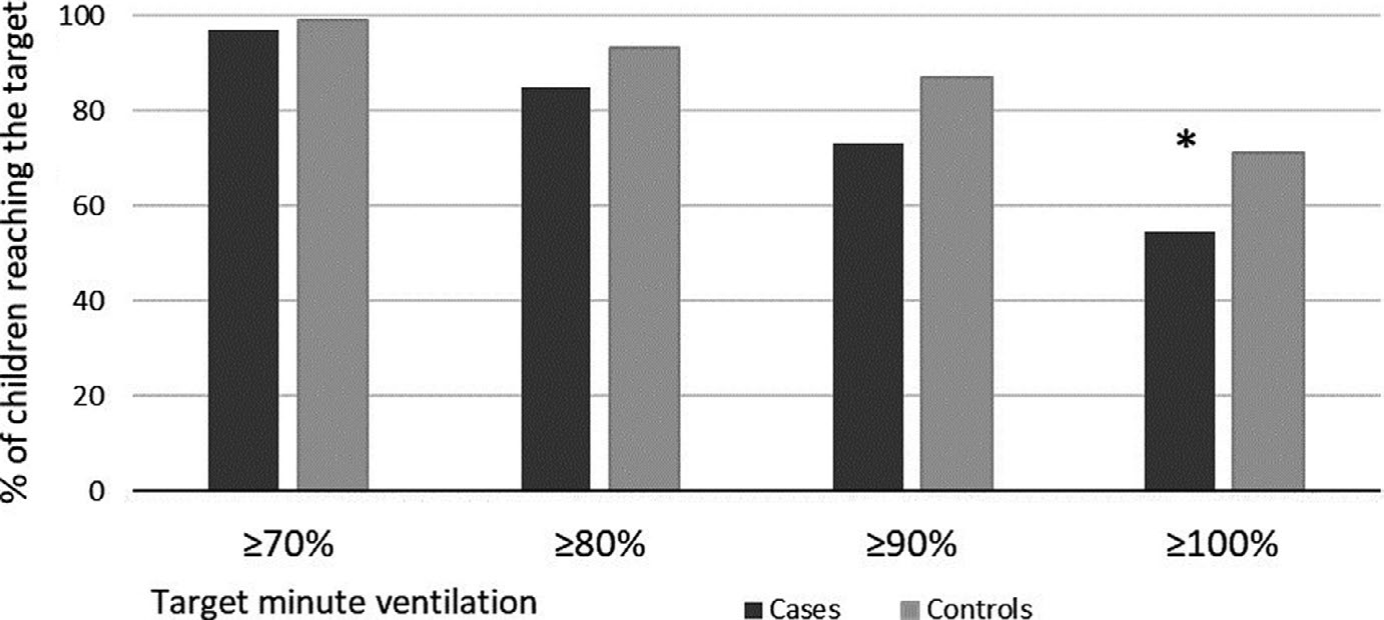
Reaching target minute ventilation volume in EVH testing. The black column presents the proportion of cases; the grey column presents the proportion of controls; *p < .05. Target minute ventilation volume defined as 30 times forced expiratory volume in 1 s

No differences were observed among cases and controls who achieved a minimum 70% of the target (130 [97%] vs. 99 [99%]; univariable *p* = .40; sex‐adjusted *p* = .52) or minimum 80% of the target (114 [85%] vs. 93 [93%]; univariable *p* = .061; sex‐adjusted *p* = .20); see Figure 2. Healthy children achieved a minimum 90% of the target (98 [73%] vs. 87 [87%]; *p* = .001; sex‐adjusted *p* = .071) and 100% of the target (73 [55%] vs. 71 [71%]; univariable *p* = .001; sex‐adjusted *p* = .017), which were generally more positive than the cases, but after adjusting for sex, the significance in which a minimum of 90% of the target was reached was lost (Figure 2). Interestingly, all 26 children who experienced bronchoconstriction reached a minimum 70% of the target minute ventilation.

### 3.4 Fall in forced expiratory volume in 1 s testing after eucapnic voluntary hyperventilation testing

Overall, the mean fall in FEV1 among all children was −4.9% (*SD* 5.7%). Bronchoconstriction, assessed as a minimum 8% fall in FEV1, occurred among 44 (19%) of all 234 children; other rates included a minimum 10% fall in FEV1 26 (11%), a minimum 12% fall in FEV1 16 (6.8%), a minimum 15% fall in FEV1 9 (3.8%) and a minimum 20% fall in FEV1 7 (3.0%). Age, sex and diagnosis of atopic eczema or asthma did not affect the fall in FEV1 after the EVH (data not shown).

**FIGURE 3:**
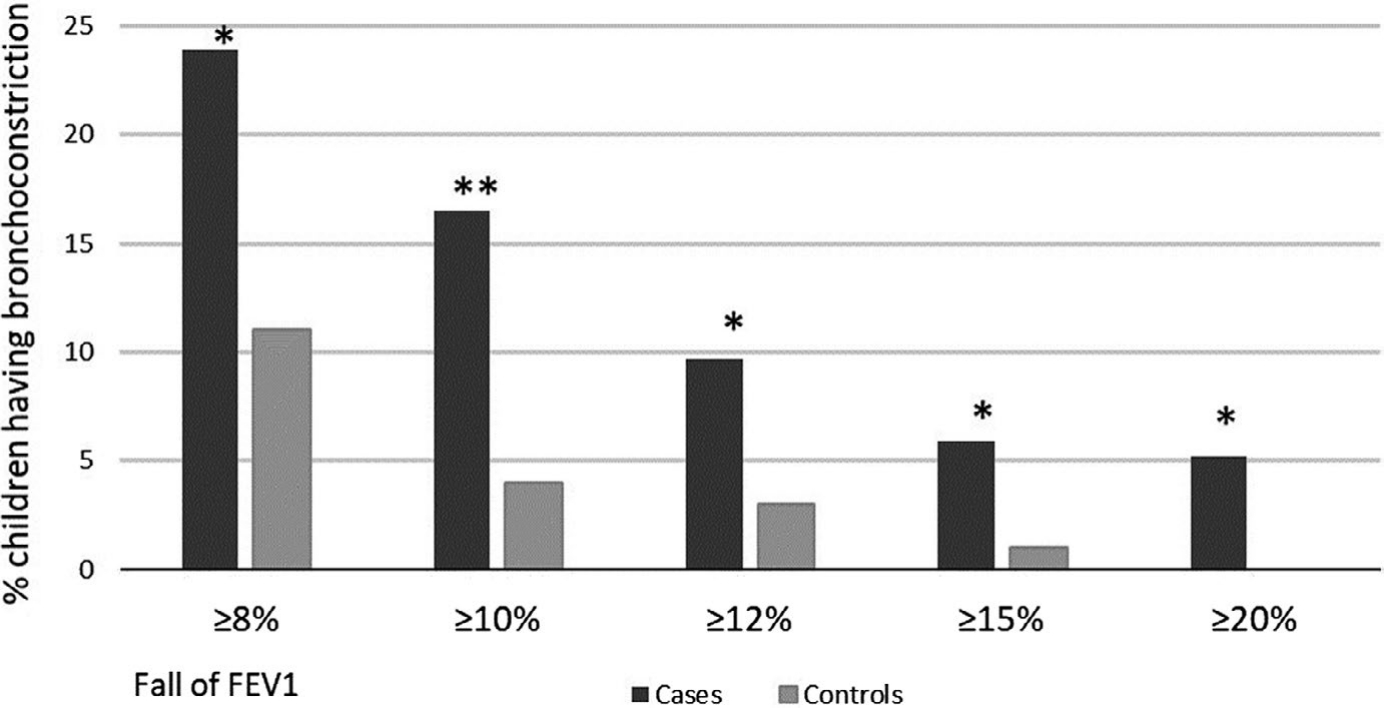
Proportion (%) of children having bronchoconstriction in different cut‐off values after EVH testing. The black column presents the proportion of cases; the grey column presents the proportion of controls; *p < .05; **p < .01. FEV1: forced expiratory volume in 1 s

A greater fall in FEV1 was observed among the cases than among the controls (mean −6.0% vs. −3.6%; *p* = .010); see Figure 3. Cases had more bronchoconstriction than controls among every cut‐off value: minimum 8% fall in FEV1: (32 [24%] versus 11 [11%], univariable *p* = .012), minimum 10% fall in FEV1: (22 [17%]) versus 4 [4%], univariable *p* = .003), minimum 12% fall in FEV1 (13 [9.7%] versus 3 [3.0%], univariable *p* = .043), minimum 15% fall in FEV1 (8 [5.9%] versus 1 [1.0%], *p* = .048) and minimum 20% fall in FEV1 (7 [5.2%] versus 0 [0%], univariable *p = *.021, Figure 3).

No differences were noted among children who reached 70%–99% of the target minute ventilation or children who reached 100% of the target in the bronchoconstriction‐related findings (Table 2). There was no correlation between reaching target minute ventilation volume and fall of FEV1 after EVH, (Spearman correlation, *r* = .061; *p* = .359).

**TABLE 2:** Test results among 229 subjects who reached a minimum 70% of target according to minute ventilation volume

Cut‐off level	Children with 70%–99% of target MVV (*n* = 87)		Children who reached 100% of target MVV (*n* = 142)	*p‐*value
10% fall of FEV1 among cases		5/58 (8.6%)	14/72 (19%)	.083
15% fall of FEV1 among cases		2/58 (3.5%)	6/72 (8.3%)	.30
10% fall of FEV1 among controls		2/29 (6.9%)	2/70 (19%)	.58
15% fall of FEV1 among controls		1/29 (3.5%)	0/70 (0.0%)	.29

Note

Data represent the number of children and the percentage. FEV1: forced expiratory volume in 1 s; MVV: minute ventilation volume. Chi‐square testing was used for statistics.

### 3.5 Cut‐off values in clinical decision‐making

When we used a cut‐off value of an 8% fall in FEV1, 32 of 43 (74%) children were cases, while using a cut‐off value of 10% fall in FEV1, 22 of 26 (85%) children were considered cases. The proportions in cut‐off values of 12% (81%) or 15% (89%) of cases were similar compared to the cut‐off value of 10%.

If a cut‐off value of 12% had been used instead of a 10% value, then there would be a 41% reduction in positive findings among the cases. A cut‐off value of 15% had a 64% reduction in positive findings among the cases, and a 20% cut‐off had a 68% reduction in positive findings among the cases (Figure 3).

### 3.6 Sensitivity and specificity in different cut‐off values to identify exercise‐induced asthma

Sensitivity and specificity in different cut‐off values are shown in Table 3.

**TABLE 3:** Sensitivity and specificity to exercise‐induced asthma in different cut‐off values

	Sensitivity (%)	Specificity (%)
Fall of FEV1 ≥ 8% after EVH	56	90
Fall of FEV1 ≥ 10% after EVH	51	98
Fall of FEV1 ≥ 12% after EVH	30	98
Fall of FEV1 ≥ 15% after EVH	19	99
Fall of FEV1 ≥ 20% after EVH	16	100
Bronchodilatation improvement ≥ 12% of FEV1 compared baseline	12	99
Bronchodilatation improvement ≥ 15% of FEV1 compared lowest value	44	96
Bronchodilatation improvement ≥ 20% of FEV1 compared lowest value	26	99

Note

EVH: eucapnic voluntary hyperventilation test; FEV1: forced expiratory volume in 1 s.

### 3.7 Bronchodilatation testing

Among all 234 children, the mean improvement in bronchodilatation testing was 2.6% (*SD* 4.5%). A minimum 12% improvement in FEV1 was observed among 7 of 234 (3.0%) children. None of the patient characteristics affected bronchodilator response. No differences were noted among cases and controls in mean improvement of bronchodilatation testing (respectively, 2.8% vs. 2.4%; univariable *p* = .062) or proportion with a minimum 12% improvement in FEV1 during bronchodilatation testing (5 [3.7%] vs. 2 [2.0%]; univariable *p* = .70). The analysis of FEV1 change in bronchodilatation test for the lowest value of FEV1 after EVH showed that the cases had greater improvement after salbutamol than controls (9.8% versus 6.4%; univariable *p* = .001). In addition, the cases had more often minimum 20% improvement in FEV1 from the lowest value of FEV1 after EVH (11 [8.3%] vs. 2 [2.0%]; univariable *p* = .045). An assessment of bronchodilatation response minimum 20% improvement from the lowest value of FEV1 after EVH compared to 12% improvement in FEV1 from the baseline improved sensitivity to EIA from 12% to 26% (Table 3).

### 3.8 Dysfunctional breathing

Of the 134 cases, 16 (12%) had objective symptoms, such as inspiratory stridor, hyperventilation or other breathing abnormalities, without significant fall of FEV1 and a lack of bronchodilator response. None of the healthy controls experienced dysfunctional breathing during EVH testing (*p* < .001).

## 4 DISCUSSION

Three main results arose from this study. First, most of the 10‐ to 16‐year‐old children successfully conducted the EVH testing without any side effects, and 70% of the target may be considered an acceptable ventilation rate. The real‐time aid of graphical and visual feedback was useful in maintaining ventilation rates. Second, a cut‐off 10% fall in FEV1 is useful for identifying those patients with exercise‐induced bronchoconstriction, and bronchodilatation testing rarely appeared to be positive after EVH testing when using a cut‐off of 12% from the baseline. Third, EVH testing is useful in identifying cases with dysfunctional breathing.

Almost all children were able to complete the EVH test at the 70% target level, regardless of any symptoms they may have experienced during the test. The subjects tolerated the EVH test well, and no additional side effects except some couching due to increased mucus production were observed among the participants. Interestingly, at high (90%–100% of the target) ventilation rates healthy children had better performance than cases. In contrast, many previous studies have shown that only the minority (range 0%–27%) of the 8‐ to 18‐year‐old children has been able to perform the test at the same 100% of target ventilation rate (Chateaubriand do Nascimento Silva Filho et al., 2015; Jara‐Gutierrez et al., 2019; Van der Eycken et al., 2016). Like in our study, the proportion of children reaching minimum 70% of target minute ventilation has been 83%–100% of 8‐ to 20‐year‐old children in previous studies (Albuquerque Rodrigues Filho et al., 2018; Chateaubriand do Nascimento Silva Filho et al., 2015; Jara‐Gutierrez et al., 2019; Van der Eycken et al., 2016). In addition, among the general adult population, 70% of the target was achieved at a much lower rate (71%) than in our study (Brummel et al., 2009). The graphical real‐time biofeedback signal data, which enabled the children to regulate their ventilation effortlessly, might have played a role in the positive results. Another key success factor might have been the research personnel's encouragement during the testing.

Children with previous asthma diagnosis had no more bronchoconstriction than children without asthma diagnosis in early childhood. This could be because many children with clinical diagnosis of asthma in early childhood had actually suffered from virus‐induced wheezing rather than “real asthma.”

In our study, a 10% cut‐off in the fall in FEV1 after EVH testing was considered optimal for the diagnosis of bronchoconstriction, because it most strikingly differentiated cases from controls. Cut‐offs of ≥10 to ≥20% had specificity to identify exercise‐induced asthma at 98%–99%, but they also markedly decreased the sensitivity from 51% to 16%, implying that if the cut‐off minimum 20% instead of 10% is used, the sensitivity would decrease significantly. Ventilation rate had no influence on this difference, which further supports the target minute ventilation of ≥70% during EVH testing among 10‐ to 16‐year‐old children. Our results are in agreement with current recommendations, according to ATS and ERS (Hallstrand et al., 2018; Parsons et al., 2013).

Bronchodilatation changes were identical in both groups but if the changes were evaluated from the lowest value in FEV1, there were significant differences between the groups. Moreover, sensitivity to identify EIA improved significantly. Results of our study are similar to a previous study done on swimmers (Romberg, Tufvesson, & Bjermer, 2012), and it may offer more relevant information compared to calculating changes from the baseline.

Eucapnic voluntary hyperventilation testing is excellent for observing dysfunctional breathing, and in our study such breathing occurred among 12% of cases and 0% of controls. This finding was in agreement with the prevalence of dysfunctional breathing, with a prevalence of 6% to 8% among the general and adolescent population (Christensen, Thomsen, Rasmussen, & Backer, 2011; Johansson et al., 2018).

The males in our study were able to reach target minute ventilation better than the females. A similar finding was also found in the largest study on EVH to have been conducted to date among the general population (Brummel et al., 2009. However, girls achieved a greater per cent predicted FEV1, which was probably the explanation for the difference**.** Age, diagnosis of atopic eczema or asthma, baseline FEV1 or fall in FEV1 after EVH did not affect the results we obtained in our study. In a previous study, a fall in FEV1 did not affect the reaching of target minute ventilation (Chateaubriand do Nascimento Silva Filho et al., 2015). Previous studies' potential confounding factors have not usually been reported.

To our knowledge, this was the largest study using EVH testing to have been conducted with children, which is a major strength of the study. One limitation of the study is that spirometry follow‐up after EVH was not made according ERS recommendations every 3 min. First spirometry follow‐up was made 1 min after EVH and fatigue might have affected first spirometry obtained. Another limitation of our study was that both cases and control children were actively engaged in sports. Their target minute ventilation achievements during EVH testing may not have been as good if the controls had been physically inactive. Our results are thus generalizable only to those who are active in sports. The proportion of males among the controls was higher compared to the cases, but sex‐adjusted analyses showed that the findings were independent of sex.

We found that EVH testing is feasible for 10‐ to 16‐year‐old children. The reaching of a minimum 70% of target minute ventilation volume may be considered acceptable performance. Another finding was that a cut‐off value of a minimum 10% fall in FEV1 also works well among children. EVH testing is also useful in identifying cases with dysfunctional breathing. Our data provide important evidence for the current ERS and ATS guidelines (Hallstrand et al., 2018; Parsons et al., 2013).

## CONFLICT OF INTEREST

The authors declare that they have no conflicts of interest.
